# Continuing trastuzumab beyond disease progression: outcomes analysis in patients with metastatic breast cancer

**DOI:** 10.1186/bcr2119

**Published:** 2008-07-16

**Authors:** Giuseppe Cancello, Emilia Montagna, Diego D'Agostino, Mario Giuliano, Antonio Giordano, Giuseppe Di Lorenzo, Monica Plaitano, Sabino De Placido, Michele De Laurentiis

**Affiliations:** 1 Dipartimento di Endocrinologia ed Oncologia Molecolare e Clinica, Università 'Federico II', via Sergio Pansini, 5, 80131 – Napoli, Italy; 2 Unità di Ricerca Senologia Medica, Istituto Europeo di Oncologia, via Ripamonti, 435, 20141-Milano, Italy

## Abstract

**Introduction:**

We performed a retrospective analysis of HER2-overexpressing metastatic breast cancer patients to describe clinical outcomes of those who, despite progression of the disease (PD), maintained trastuzumab for multiple chemotherapy lines. We also compared survival of these patients with that of those who halted trastuzumab at first PD.

**Methods:**

We identified 101 patients treated between July 2000 and January 2007. Nineteen were still receiving the first-line trastuzumab-based treatment without evidence of PD and were not included in this analysis. Of the remaining 82 patients, 59 retained trastuzumab for one or more additional lines of chemotherapy after PD, according to our institution policy. Twenty-three patients who changed treating institution and stopped trastuzumab at first progression were used as a control group.

**Results:**

For patients retaining trastuzumab, the median follow-up was 39.6 months. Clinical outcomes showed the typical degradation between first and second lines of therapy which we would expect by halting trastuzumab at first progression. Response rates were 35% and 16% and median times to progression were 7.25 and 5.25 months for the first and second lines of trastuzumab therapy, respectively. The median overall survival (OS) rates were 70 months for patients who retained trastuzumab and 56 months for patients who halted the drug (hazard ratio [HR] 0.87, 95% confidence interval [CI] 0.51 to 1.18; *P *= 0.52). If we consider OS from the start of trastuzumab therapy, the figures are 53.9 and 34.8 months, respectively (HR 0.78, 95% CI 0.58 to 1.32; *P *= 0.2).

**Conclusion:**

A nonstatistically significant trend of improved survival for patients retaining trastuzumab is observed. This is in line with most retrospective analyses and recent randomized data. Retaining trastuzumab after progression is a reasonable option, but further randomized data are warranted to better define its role in comparison with other available options.

## Introduction

Trastuzumab is a humanized anti-HER2 monoclonal antibody directed against the HER2 protein (p185HER2/neu), which is the product of the HER2 proto-oncogene (also designated as c-erbB-2 or HER2/neu). HER2 is overexpressed in approximately 20% to 25% of breast tumors [1,2]. This alteration is associated with poor prognosis and may affect the response to hormonal therapy and chemotherapy [3,4]. Trastuzumab demonstrated a benefit as a single agent in first- or second-line treatment of HER2-overexpressing (HER2^+^) metastatic breast cancer (MBC) [5,6]. Furthermore, in two randomized trials involving chemotherapy-naive HER2^+ ^MBC patients, trastuzumab in combination with chemotherapy yielded a longer time to disease progression and a longer median survival as compared with chemotherapy alone [7,8]. According to these results, trastuzumab has been approved for use in combination with taxanes as first-line treatment of HER2^+ ^MBC and its administration is usually allowed after completion of chemotherapy up to the progression of the disease (PD), the time when trastuzumab should be withdrawn and a second-line chemotherapy started (see regulatory approval by the Food and Drug Administration in the US or by the European Medicines Agency in Europe).

The choice to stop trastuzumab at disease progression indirectly derives from the traditional use of cytotoxic treatments, whose discontinuation upon disease progression is warranted because both there is no experimental evidence of a benefit in continuing the same treatment and overlapping toxicity does not permit concomitant delivery of non-crossresistant cytotoxic agents. It has been argued, however, that this paradigm may not apply to molecularly targeted drugs like trastuzumab. For instance, preclinical data suggest that continuation of treatment with trastuzumab is required for sustained tumor control in breast cancer in nude mice [9], thus supporting treatment extension after first-line progression. Moreover, although the exact mechanism of action of this drug is unclear, preclinical models indicate a synergistic antitumoral effect between trastuzumab and chemotherapy [10]. Therefore, some authors have hypothesized that trastuzumab administration should continue despite disease progression in order to take advantage of a potential synergistic interaction with second-line chemotherapy. More recent preclinical experimental data are contradictory in this regard. Tripathy and colleagues [11] suggest that breast cancer cell proliferation is inhibited partially by continuing trastuzumab treatment even after the development of resistance to the drug. Similarly, Shirane and colleagues [12] show that retaining trastuzumab therapy improves the cytotoxic effect of taxanes against trastuzumab-resistant xenografts in nude mice. Conversely, Nahta and colleagues [13] report that the continued exposure to trastuzumab after development of resistance did not improve the efficacy of the chemotherapeutic drug vinorelbine.

From a clinical standpoint, relevant data are scanty and contradictory [14-21] and therefore whether to continue the administration of trastuzumab beyond disease progression remains an unanswered question. To help resolve this issue, we present a retrospective analysis of the series of patients treated with trastuzumab at our institution, in order to describe characteristics and clinical outcomes of patients who halted the treatment at first evidence of progression as compared with those who were maintained on trastuzumab in spite of the PD.

## Materials and methods

We searched clinical and pharmacy records of the Oncology Division at the University of Naples 'Federico II', Italy, to identify patients with HER2^+ ^MBC who had received at least one line of trastuzumab therapy between July 2000 and January 2007. The policy adopted at our institution in that period was to continue trastuzumab after progression for multiple lines of chemotherapy, if medically feasible. After first progression, however, some patients decided to receive further therapies at other institutions and usually were put off trastuzumab. Because their decision was apparently based on nonmedical reasons, we followed them up by telephone calls and used their survival data as a control in this analysis. All patients who retained trastuzumab therapy after progression gave their written informed consent to the off-label use of the drug. Trastuzumab was administered according to either the standard weekly regimen (loading dose of 4 mg/kg and then 2 mg/kg weekly) or the 3-weekly regimen (loading dose of 8 mg/kg and then 6 mg/kg every 3 weeks). HER2 expression was assessed by immunohistochemistry with the polyclonal Dako test (Dako, Glostrup, Denmark) and scored for the majority of patients as 3+ or 2+. For tumors scoring 2+, a fluorescence *in situ *hybridization test was mandatory to confirm HER2 gene amplification. Some patients were tested by a monoclonal antibody (CB11) and were declared HER2^+ ^if they exhibited intense and diffuse membrane staining in more than 10% of tumor cells. Tumor response was evaluated by imaging procedures such as computed tomography scan or magnetic resonance imaging or by clinical examination. Tumor response was based on RECIST (Response Evaluation Criteria in Solid Tumors) criteria, when applicable. In the absence of measurable lesions, the disease was considered stable unless a clinical progression was identified based on the judgment of the treating physician.

### Statistical analysis

Objective response rate (ORR) was defined as the sum of complete response and partial response (CR + PR). Clinical benefit was defined as the sum of ORR and stable disease lasting at least 24 weeks (CR + PR + SD). Time to progression (TTP) was defined as the time from the start of each treatment with trastuzumab to the date of documented progression. Overall survival (OS) was defined as the time from the date of diagnosis of metastatic disease until death from any cause. Since some patients were not treated with trastuzumab as the first line of therapy for metastatic disease, we also calculated trastuzumab-start OS (tsOS), defined as the time from the initiation of trastuzumab treatment until death. To reduce further the bias derived from the effect of trastuzumab first-line therapy, we also calculated post-progression OS (ppOS), defined as the time from the first documented progression after the trastuzumab therapy until death from any cause. For OS, tsOS, and ppOS, surviving patients were censored at the time of their last follow-up. OS, tsOS, and ppOS were estimated by the Kaplan-Meier method. All *P *values were two-sided and were considered significant if their value was less than 0.05.

## Results

One hundred one patients with trastuzumab-treated MBC were identified. Of these, 19 were still receiving the first line of trastuzumab-based therapy without progression and therefore are not included in the present analysis. Among the remaining 82 patients, 23 had stopped trastuzumab to the first progression because they elected to continue therapy elsewhere, whereas 59 had retained it for one or more additional lines of chemotherapy.

### Patient characteristics

Characteristics of patients according to whether trastuzumab was halted or retained upon progression are summarized in Table 1. Trastuzumab-based treatment was used as first-line therapy for 35 (59%) patients retaining trastuzumab and for 12 (52%) patients halting trastuzumab at progression. Most patients received trastuzumab in combination with a single chemotherapeutic agent. The most frequently administered cytotoxic drugs were taxanes (docetaxel or paclitaxel) and then vinorelbine in both groups of patients and for either first or subsequent treatment lines. Among the 59 patients retaining trastuzumab, 27 kept on trastuzumab up to a third line of chemotherapy and 14 up to a fourth line. Overall, there is no statistically significant difference in the distribution of main patient characteristics between the two groups. However, a nonstatistically significant imbalance was observed for age of less than 50 years, poorly differentiated (G3) tumors, adjuvant administration of anthracycline ± taxanes, and involvement of at least two metastatic sites, which were all more frequent among patients retaining trastuzumab.

**Table 1:** Patient characteristics

	Patients retaining trastuzumab		Patients halting trastuzumab	
	Percentage	Number 59	Percentage	Number 23

Age in years, mean (range)^a^	51 (26–74)		58 (36–77)	
<50 years	41	24	22	5
≥ 50 years	59	35	78	18
Oestrogen receptor/Progesterone receptor				
+/+	49	29	43	10
+/-	10	6	9	2
-/-	39	23	39	9
Unknown	2	1	9	2
Node				
Negative	12	7	13	3
Positive	66	39	52	12
Unknown	22	13	35	8
Grade				
1–2	10	6	26	6
3	54	32	43	10
Unknown	36	21	31	7
Adjuvant Therapy				
Anthracycline	44	26	31	7
Anthracycline/taxane	10	6	13	3
No anthracycline/taxane	46	27	56	13
HER2				
2+^b^	12	7	9	2
3+	56	33	48	11
Other (positive)^c^	32	19	43	10
Site of disease before trastuzumab therapy^d^				
1 site	59	35	78	18
≥ 2 sites	41	24	22	5
Bone		23		5
Liver		14		7
Lung		15		7
Nodes		9		2
Skin		9		4
Other		13		3

^a^Age at the time when trastuzumab therapy was started. ^b^All 2+ patients had amplification of HER2 gene (fluorescence *in situ *hybridization-positive). ^c^Positive was indicated by a pathologist as intense and diffuse membrane staining in more than 10% of tumor cells. ^d^Site information refers to the start of trastuzumab treatment.

### Clinical outcomes

**Figure 1:**
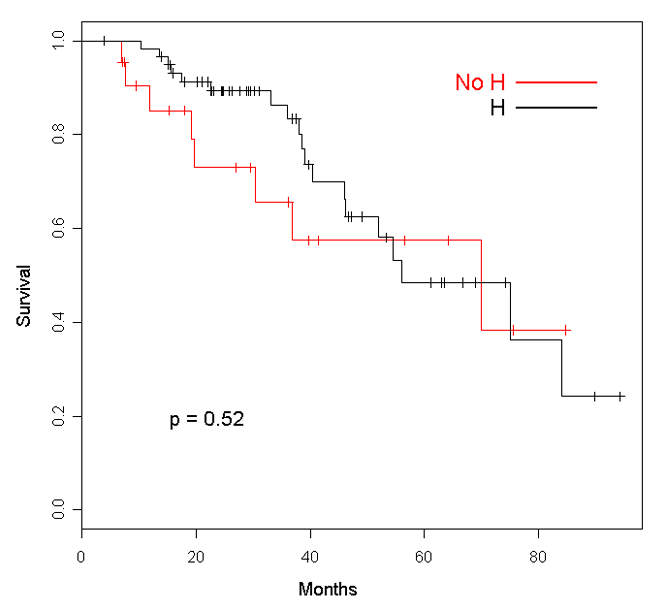
Overall survival of patients retaining trastuzumab (H) and patients halting trastuzumab (No H).

**Figure 2:**
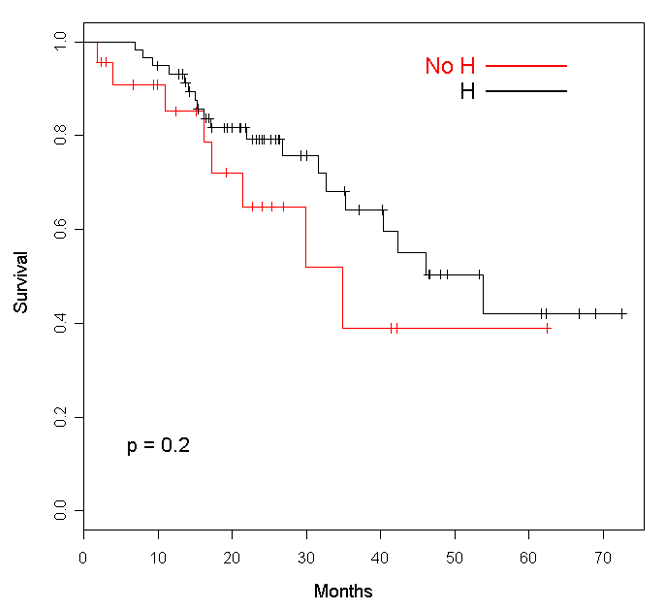
Overall survival from the start of trastuzumab of patients retaining (H) and patients halting (No H) trastuzumab.

**Figure 3:**
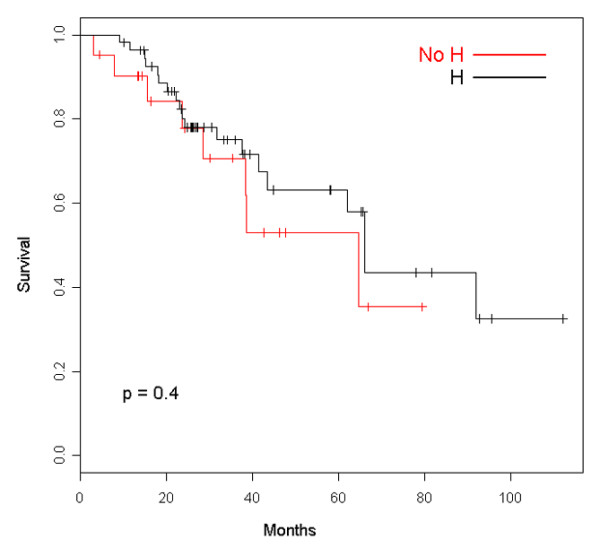
Overall survival from the first documented progression after the trastuzumab therapy of patients retaining (H) and patients halting (No H) trastuzumab.

Clinical outcomes of patients retaining trastuzumab beyond disease progression are summarized in Table 2. The median follow-up was 39.6 months (95% confidence interval [CI] 29.5 to 56.5 months), and the median duration of trastuzumab therapy was 16.5 months. As can be seen, after the first line of treatment, ORR, clinical benefit, and TTP show the typical deterioration that is traditionally observed with chemotherapy alone. Most patients halting trastuzumab at first PD were not evaluable for tumor response assessment because they usually continued treatment at other institutions. However, we were able to follow them up for survival data by telephone calls. We have recorded 20 and 8 deaths in the groups retaining and halting trastuzumab, respectively. Figure 1 depicts the OS curve for both groups, showing a nonstatistically significant trend of improved survival for patients continuing the drug after progression (hazard ratio [HR] 0.87, 95% CI 0.58 to 1.32). A similar nonsignificant trend favoring patients who retained trastuzumab was observed for tsOS (Figure 2) with an HR of 0.78 (95% CI 0.51 to 1.18). Where ppOS was concerned, there is also a trend in favor of the group continuing trastuzumab beyond PD, though nonstatistically significant (HR 0.85, 95% CI 0.56 to 1.29) (Figure 3). Safety was not a primary objective of this retrospective analysis; however, it is worth noting that only one symptomatic cardiac event was recorded after first-line therapy for patients keeping on trastuzumab.

**Table 2:** Clinical outcomes of trastuzumab beyond disease progression

Median follow-up 39.6 months				
Median duration of trastuzumab therapy 16.5 months				

	First-line trastuzumab (n = 57)	Second-line trastuzumab (n = 55)	Third-line trastuzumab (n = 26)	Fourth-line trastuzuamb (n = 12)

Objective response rate				
CR + PR, (number) percentage	(20)^a ^35	(9)^b ^16	(4)^c ^15	(0) 0
CR, (number) percentage	(6) 10.5	(2) 3.5	(0) 0	(0) 0
PR, (number) percentage	(14) 24.5	(7) 12.5	(4) 15	(0) 0
Clinical benefit, percentage (CR + PR + SD)	74 (6 + 14 + 22)	53 (2 + 7 + 20)	60 (0 + 4 + 11)	17 (0 + 0 + 2)
Time to progression in months, median (range)	7.25 (1.5–46.5)	5.25 (1.25–34.2)	5.25 (1–33.5)	3.75 (1–7)

^a^Two patients were not evaluable. ^b^Four patients were not evaluable. ^c^One patient was not evaluable. CR, complete response; PR, partial response; SD, stable disease.

## Discussion

Trastuzumab has dramatically changed the treatment of HER2^+ ^MBC. Its combination with chemotherapy is the mainstay of first-line treatment of breast tumors with HER2 amplification/overexpression. According to the regulatory approval in the US and Europe, trastuzumab should be administered up to the first evidence of PD. Many clinicians, however, prolong the administration of the drug for various chemotherapy lines in the attempt to exploit a potential synergism with further chemotherapy regimens. However, this 'off-label' practice, which has never been proven to be effective, has important drawbacks, such as a dramatic increase of treatment-related costs and a potential yield of treatment-related adverse events.

From a safety perspective, however, available data seem reassuring. In our study, patients were routinely monitored for cardiac events by clinical examination and every cycle evaluation of left ventricular ejection fraction. We recorded only one symptomatic cardiac event during prolonged administration of trastuzumab after first PD, which is in line with the results of the H0659g trial, a large extension trial specifically designed to evaluate the safety profile of trastuzumab beyond disease progression. In the H0659g trial, indeed, no new specific adverse events were seen with any particular chemotherapy regimen or with prolonged administration of trastuzumab up to 40 months. No cumulative toxicities emerged and cardiac dysfunction was uncommon, suggesting that the long-term trastuzumab treatment is well tolerated and that the development of trastuzumab-associated cardiac dysfunction is not determined by the duration of trastuzumab treatment or by the cumulative dose of the drug [22].

From an efficacy perspective, we were unable to lend clear support to this 'off-label' use of trastuzumab. This is based particularly on the observation that patients retaining trastuzumab for multiple chemotherapy lines experienced, in our series, a progressive worsening of treatment-related outcomes. This appears similar to what we would expect with chemotherapy alone, thus casting doubts on the potential advantage of keeping the patients on trastuzumab after the first progression. Various authors have carried out similar analyses showing heterogeneous results. As far as response rate is concerned, many authors reported results that are analogous to ours [14,17,19,20], but in two studies [18,21] response rates have appeared very similar for first- and second-line trastuzumab-based treatments (Table 3), thus supporting the hypothesis of a potentiation of further chemotherapy regimens by retaining trastuzumab after progression. When TTP is analyzed, studies have again shown inconsistent results (Table 3). In our study, the median TTP was 2.0 months shorter for second-line treatment as compared with first-line. A similar TTP degradation is reported by Montemurro and colleagues [20] (median TTP: 9.0 versus 6.3 months, first versus second line, respectively) and by Metro and colleagues [19] (median TTP: 9.5 versus 6.7 months, first versus second line, respectively). Conversely, in the Spanish and Austrian studies [14,17], TTP is approximately constant between the first and second lines, whereas in the global retrospective analysis by Gelmon and colleagues [18], the TTP of the second trastuzumab regimen is even longer than the first one (26 versus 23.5 weeks, respectively). At analytical examination, we were unable to identify distinguishing characteristics of these studies which may explain such different results. However, it has to be underscored that this kind of study is prone to many biases. For instance, the reported outcomes for the trastuzumab-based regimens may have been heavily influenced by several factors, like the kind of chemotherapy used and the characteristics of patients (that is, prior chemotherapy exposure, disease-free interval, tumor burden, dominant metastatic site, and so on). Therefore, any combination of results for first- and second-line treatments may have been observed just for the play of such factors or by chance, making it unreasonable to draw conclusions or hypotheses based on these data.

**Table 3:** Summary of outcomes reported of trastuzumab beyond disease progression series

Study by country	Patients, number		ORR, percentage		CBR, percentage		TTP, months		OS, months	
	First	Second	First	Second	First	Second	First	Second		
	HaltT	RetT							HaltT	RetT
Global [18]	77	65	39	32	69	54	5.5	6.1		
		105							nr	29
Hellenic [16]		80	nr	24	nr	52	nr	5.2		
		80							nr	22.2
German [14]	23	23	56.6	39.1	nr	nr	nr	nr		
	113	23							38.5^a^	62.4^a^
Austrian [21]	54	nr	42.6	25.9	85.2	68.5	6.0	6.0		
									nr	nr
Spanish [17]	93	47	46.6	29.8	71	51.1	5.0	4.0		
									nr	nr
French [15]			nr	nr	nr	nr	nr	nr		
	70	107							4.6^b^	NR^b^
Italian 1 [20]	184	40	55.4	17.9	nr	nr	9.0	6.3		
	71	40							30.2^b^	30.1^b^
Italian 2 [19]	59	37	59.3	27	83	62.2	9.5	6.7		
									nr	nr
Italian 3	57	55	36	17	73	55	7.25	5.25		
	23	59							29.9^b^	42.3^b^

^a^OS is calculated from the diagnosis of metastatic disease to the documented date of death. ^b^OS is calculated from the day of the first administration of trastuzumab-based therapy to the documented date of death. CBR, clinical benefit rate; HaltT, patients halting trastuzumab; nr, not reported; NR, not reached after median follow-up of 27.8 months; ORR, objective response rate; OS, overall survival; RetT: patients retaining trastuzumab; TTP, time to progression.

A more informative approach is to compare the survival of patients who maintained trastuzumab with that of patients who stopped the drug upon first progression, provided that no major patient characteristic has driven the treatment choice. Only four studies, including ours, reported such data, with three of them showing a longer survival for patients who maintained trastuzumab. In the Italian study by Montemurro and colleagues [20], survival of patients who continued or discontinued trastuzumab is practically identical. In the German study [21], a statistically significant longer survival for patients who received two or more trastuzumab-based regimens is reported. In a French study presented recently at the San Antonio Breast Cancer Symposium, survival of patients maintaining trastuzumab is much longer than for patients halting the drug [15]. However, this difference is so large that a selection bias may have occurred. For instance, if patients halting trastuzumab include those who are unfit to receive further chemotherapy, the observed difference may well be explained by the bad prognosis of these patients; unfortunately, this issue is not clarified by the authors. Moreover, in the French study, patients who retained trastuzumab would seem to have a longer median disease-free interval, less visceral tumor burden, and (above all) fewer poorly differentiated tumors; so these features could have contributed to the survival advantage for patients continuing trastuzumab.

In our study, we have found a trend toward a better survival for patients continuing trastuzumab beyond progression; this trend increases when we calculate OS from the start of trastuzumab therapy. Although statistical significance is not reached in our analysis, we believe that these results support a possible advantage in continuing trastuzumab beyond disease progression. Indeed, our study is relatively small and the lack of a formal statistical significance may well be ascribed to the low statistical power of the study. Furthermore, in our series, patients halting trastuzumab after PD were those who elected to continue treatment at other institutions for nonmedical reasons. Therefore, a major selection bias favoring survival for these patients should not have occurred. Quite the opposite, in our study, patients who continued trastuzumab were younger, had tumors that were less differentiated, had received more adjuvant chemotherapy, and (finally) had a higher tumor burden (Table 1). Although these differences are not statistically significant, they theoretically could have diluted a beneficial effect of retaining trastuzumab.

Lapatinib is a tyrosine-kinase inhibitor that has been shown to improve the efficacy of capecitabine for HER2^+ ^patients progressing on trastuzumab-based therapy and recently has been granted approval for the treatment of such patients. For two reasons, however, the availability of this drug does not limit the interest in the off-label use of trastuzumab after progression. First, the study by Geyer and colleagues [23] did not show any survival advantage for patients receiving lapatinib. Although survival was not the primary endpoint, the lack of a survival improvement remains unexplained because in that study a crossover to lapatinib after progression was not allowed to patients who had received capecitabine alone. A possible explanation is that the advantage of getting lapatinib is temporary and is completely offset by a shorter ppOS for patients randomly assigned to lapatinib. Second, if the 'principle' that continuing trastuzumab after progression is proven effective, reinstating trastuzumab therapy even after progression on lapatinib could be a valid option [23].

Three ongoing randomized trials are directly evaluating the efficacy of continuing trastuzumab after progression. A European multicenter randomized phase III study coordinated by the German Breast Cancer Group, namely the Trastuzumab Beyond Progression (TBP) trial, is comparing capecitabine alone versus the combination capecitabine-trastuzumab upon progression of a previous treatment with trastuzumab [24]. The trial started recruitment on June 2003 aiming at a target of 482 patients, but because of a low accrual the trial was closed at 156 patients. A more pragmatic approach is followed in the other two trials: the 'Pandora' study, which is an international European study sponsored by F. Hoffmann-La Roche Ltd. (Basel, Switzerland) [25], and the recently started THOR (Trastuzumab Halted Or Retained) trial, which is an Italian study coordinated at our institution and sponsored by F. Hoffmann-La Roche Ltd. [26]. The main difference of these pragmatic studies compared with the German trial is that second-line chemotherapy is not fixed but investigators can choose among numerous regimens enlisted in the protocol. Quite recently, results from the TBP trial have been presented [27], showing that keeping trastuzumab in combination with capecitabine after progression yields a statistically significant improvement of TTP (8.2 versus 5.6 months; *P *= 0.03), but not of OS (25.5 versus 20.4 months; *P *= 0.26). It remains to be defined whether other chemotherapy regimens benefit from trastuzumab continuation. Also, to check whether survival is improved by continuing trastuzumab will require a longer follow-up, additional data, or both.

## Conclusion

In our analysis, we observed a nonstatistically significant trend of improved survival for patients retaining trastuzumab with multiple lines of treatment. These results are in agreement with most, but not all, retrospective data and provide additional support to preliminary randomized data showing an improvement of TTP. Additional randomized data are needed to better define the role of continuing trastuzumab after progression, with particular regard to the eventual OS improvement. Meanwhile, the 'off-label' practice of continuing trastuzumab after progression stands as a reasonable therapeutic option, particularly for patients who are intolerant to lapatinib.

## Abbreviations

CI = confidence interval; CR = complete response; HER2^+ ^= HER2-overexpressing; HR = hazard ratio; MBC = metastatic breast cancer; ORR = objective response rate; OS = overall survival; PD = progression of the disease; ppOS = post-progression overall survival; PR = partial response; TBP = Trastuzumab Beyond Progression; tsOS = trastuzumab-start overall survival; TTP = time to progression.

## Competing interests

MDL and SDP have received speaking honoraria from F. Hoffmann-La Roche Ltd. (Basel, Switzerland). Dipartimento di Endocrinologia ed Oncologia Molecolare e Clinica, Università 'Federico II', the affiliation of all of the authors except EM, is the leading institution of the THOR (Trastuzumab Halted Or Retained) randomized trial, which is sponsored by F. Hoffmann-La Roche Ltd. The other authors declare that they have no competing interests.

## Authors' contributions

GC and MDL shared responsibility for the conception and design of the study and for the analysis and interpretation of data and helped to draft the manuscript. EM shared responsibility for the acquisition and interpretation of data and helped to draft the manuscript. MG and AG shared responsibility for the acquisition and interpretation of data. DDA performed the statistical analysis and contributed to the interpretation of data. SDP and GDL helped to give final approval of the version to be submitted. All authors revised the manuscript critically for important intellectual content and read and approved the final manuscript.

## References

[B1] Slamon DJ, Clark GM, Wong SG, Levin WJ, Ullrich A, McGuire WL (1987). Human breast cancer: correlation of relapse and survival with amplification of the HER-2/neu oncogene. Science.

[B2] Slamon DJ, Godolphin W, Jones LA, Holt JA, Wong SG, Keith DE, Levin WJ, Stuart SG, Udove J, Ullrich A (1989). Studies of the HER-2/Neu proto-oncogene in human breast and ovarian cancer. Science.

[B3] Ross JS, Fletcher JA, Linette GP, Stec J, Clark E, Ayers M, Symmans WF, Pusztai L, Bloom KJ (2003). The Her-2/neu gene and protein in breast cancer 2003: biomarker and target of therapy. Oncologist.

[B4] De Laurentiis M, Arpino G, Massarelli E, Ruggiero A, Carlomagno C, Ciardiello F, Tortora G, D'Agostino D, Caputo F, Cancello G, Montagna E, Malorni L, Zinno L, Lauria R, Bianco AR, De Placido S (2005). A meta-analysis on the interaction between HER-2 expression and response to endocrine treatment in advanced breast cancer. Clin Cancer Res.

[B5] Cobleigh MA, Vogel CL, Tripathy D, Robert NJ, Scholl S, Fehrenbacher L, Wolter JM, Paton V, Shak S, Lieberman G, Slamon DJ (1999). Multinational study of the efficacy and safety of humanized anti-HER2 monoclonal antibody in women who have HER2-overexpressing metastatic breast cancer that has progressed after chemotherapy for metastatic disease. J Clin Oncol.

[B6] Vogel CL, Cobleigh MA, Tripathy D, Gutheil JC, Harris LN, Fehrenbacher L, Slamon DJ, Murphy M, Novotny WF, Burchmore M, Shak S, Stewart SJ, Press M (2002). Efficacy and safety of trastuzumab as a single agent in first-line treatment of HER2-overexpressing metastatic breast cancer. J Clin Oncol.

[B7] Marty M, Cognetti F, Maraninchi D, Snyder R, Mauriac L, Tubiana-Hulin M, Chan S, Grimes D, Antón A, Lluch A, Kennedy J, O'Byrne K, Conte P, Green M, Ward C, Mayne K, Extra JM (2005). Randomized phase II trial of the efficacy and safety of trastuzumab combined with docetaxel in patients with human epidermal growth factor receptor 2-positive metastatic breast cancer administered as first-line treatment: the M77001 study group. J Clin Oncol.

[B8] Slamon DJ, Leyland-Jones B, Shak S, Fuchs H, Paton V, Bajamonde A, Fleming T, Eiermann W, Wolter J, Pegram M, Baselga J, Norton L (2001). Use of chemotherapy plus a monoclonal antibody against HER2 for metastatic breast cancer that overexpresses HER2. N Engl J Med.

[B9] Pietras RJ, Pegram MD, Finn RS, Maneval DA, Slamon DJ (1998). Remission of human breast cancer xenografts on therapy with humanized monoclonal antibody to HER-2 receptor and DNA-reactive drugs. Oncogene.

[B10] Pegram M, Hsu S, Lewis G, Pietras R, Beryt M, Sliwkowski M, Coombs D, Baly D, Kabbinavar F, Slamon D (1999). Inhibitory effects of combinations of HER-2/neu antibody and chemotherapeutic agents used for treatment of human breast cancers. Oncogene.

[B11] Tripathy D, Hassan S, Verma S, Gurnani P, Nandi A, Rosenblatt K (2005). Phenotypic and proteomic alterations of acquired trastuzumab resistance. J Clin Oncol.

[B12] Shirane M, Fujimoto-Ouchi K, Sekiguchi F, Nunomura K, Mori K (2005). Preclinical study of continuous administration of trastuzumab as combination therapy after disease progression with trastuzumab monotherapy. Eur J Cancer.

[B13] Nahta R, Esteva FJ (2004). *In vitro *effects of trastuzumab and vinorelbine in trastuzumab-resistant breast cancer cells. Cancer Chemother Pharmacol.

[B14] Bartsch R, Wenzel C, Hussian D, Pluschnig U, Sevelda U, Koestler W, Altorjai G, Locker GJ, Mader R, Zielinski CC, Steger GG (2006). Analysis of trastuzumab and chemotherapy in advanced breast cancer after the failure of at least one earlier combination: an observational study. BMC Cancer.

[B15] Extra J-M, Antoine E-C, Vincent-Salomon A, Bergougnoux L, Campana F, Namer M (2006). Favourable effect of continued trastuzumab treatment in metastatic breast cancer patients: results from the French Hermine cohort study. Breast Cancer Res Treat.

[B16] Fountzilas G, Razis E, Tsavdaridis D, Karina M, Labropoulos S, Christodoulou C, Mavroudis D, Gogas H, Georgoulias V, Skarlos D (2003). Continuation of trastuzumab beyond disease progression is feasible and safe in patients with metastatic breast cancer: a retrospective analysis of 80 cases by the hellenic cooperative oncology group. Clin Breast Cancer.

[B17] Garcia-Saenz J, Martin M, Bueno C, Sampedro T, Lopez-Tarruella S, Puente J, Villalobos L, Rodriguez L, Garcia B, Casado A, Diaz-Rubio E (2005). Trastuzumab associated with successive cytotoxic therapies beyond disease progression in metastatic breast cancer. Clin Breast Cancer.

[B18] Gelmon KA, Mackey J, Verma S, Gertler SZ, Bangemann N, Klimo P, Schneeweiss A, Bremer K, Soulieres D, Tonkin K, Bell R, Heinrich B, Grenier D, Dias R (2004). Use of trastuzumab beyond disease progression: observations from a retrospective review of case histories. Clin Breast Cancer.

[B19] Metro G, Mottolese M, Di Cosimo S, Papaldo P, Ferretti G, Carlini P, Cianciulli AM, Giannarelli D, Cognetti F, Fabi A (2007). Activity of trastuzumab (t) beyond disease progression in HER2 over-expressing metastatic breast cancer (MBC). J Clin Oncol.

[B20] Montemurro F, Donadio M, Clavarezza M, Redana S, Jacomuzzi ME, Valabrega G, Danese S, Vietti-Ramus G, Durando A, Venturini M, Aglietta M (2006). Outcome of patients with HER2-positive advanced breast cancer progressing during trastuzumab-based therapy. Oncologist.

[B21] Stemmler HJ, Kahlert S, Siekiera W, Untch M, Heinrich B, Heinemann V (2005). Prolonged survival of patients receiving trastuzumab beyond disease progression for HER2 overexpressing metastatic breast cancer (MBC). Onkologie.

[B22] Tripathy D, Slamon DJ, Cobleigh M, Arnold A, Saleh M, Mortimer JE, Murphy M, Stewart SJ (2004). Safety of treatment of metastatic breast cancer with trastuzumab beyond disease progression. J Clin Oncol.

[B23] Geyer CE, Forster J, Lindquist D, Chan S, Romieu CG, Pienkowski T, Jagiello-Gruszfeld A, Crown J, Chan A, Kaufman B, Skarlos D, Campone M, Davidson N, Berger M, Oliva C, Rubin SD, Stein S, Cameron D (2006). Lapatinib plus capecitabine for HER2-positive advanced breast cancer. N Engl J Med.

[B24] GBG German Breast Group, Herceptin – TBP [in German]. http://www.germanbreastgroup.de/herceptin.

[B25] A Study of Herceptin (Trastuzumab) in Combination with 2nd-Line Chemotherapy in Patients with HER2 Positive Metastatic Breast Cancer. http://www.cancer.gov/search/ViewClinicalTrials.aspx?cdrid=539603&version=HealthProfessional&protocolsearchid=3577090.

[B26] THOR Study: A Study of Continued Herceptin (Trastuzumab) in Combination With Second Line Chemotherapy in Patients With HER2 Positive Metastatic Breast Cancer. http://www.cancer.gov/search/ViewClinicalTrials.aspx?cdrid=542712&version=HealthProfessional&protocolsearchid=3577090.

[B27] Von Minckwitz G, Zielinski C, Maarteense E, Vogel P, Schmidt M, Eidtmann H, Cufer T, de Jongh FE, Kaufmann M, Loibl S (2008). Capecitabine vs. capecitabine + trastuzumab in patients with HER2-positive metastatic breast cancer progressing during trastuzumab treatment: The TBP phase III study (GBG 26/BIG 3-05) [abstract]. J Clin Oncol.

